# Supplementary material for: Reconciling larval and adult sampling methods to model growth across life-stages
Source: PLoS One. 2020 Aug 21;15(8):e0237737. doi: 10.1371/journal.pone.0237737 (PMC7442236; doi:10.1371/journal.pone.0237737)
Supplement: Attachment — Submitted filename: PONE-D-20-12247_Review.docx [file pone.0237737.s002.docx]

**Review of PONE-D-20-12247, “Reconciling larval and adult sampling methods to model growth across life-stages,” by George C. Brooks, Thomas A. Gorman, Yan Jiao, and Carola A. Haas.**

General Comments:

This manuscript addresses two important issues related to modeling population growth of organisms with complex life-histories: 1) the inability to accurately age individuals of many species, and 2) quantifying changes in growth across life stages of such organisms. The manuscript presents a model that provides estimates of parameters from larval growth through metamorphosis to adult growth. Such a model is very appealing, because it allows estimation of growth parameters across life-history stages to encompass an organism’s entire lifespan. Being able to model growth across life stages can be particularly important for parameterizing population models to develop conservation strategies. Thus, the topic of this manuscript is both interesting and important.

Although generally well-written, the organization of the manuscript could be improved. For example, although the Introduction nicely presents the larger context of modeling growth in species with complex life histories and presents information on the study species, it fails to concisely state the objectives of the study. The Introduction also contains a paragraph describing methods; the Materials and Methods then refer the reader back to the Introduction for this information. I recommend moving the methods paragraph from the Introduction to the Materials and Methods and altogether avoiding the awkward reference. In addition to this reorganization, some material based entirely on unpublished data or personal communications could be removed from the Discussion.

Additional information is necessary in the Materials and Methods. I was left wondering how often the breeding wetlands were sampled, what time of year they were sampled, whether all wetlands were sampled each year (and if not, how many were sampled each year), how many times each year they were sampled, whether the larvae were marked (which could be valuable for a growth study even if the mark isn’t retained through metamorphosis), etc. Even less information was presented about adults—all the reader can tell is that they were sampled using drift fences (and presumably pit fall traps). When were the traps active, and how did this relate to the timing of breeding, larval sampling, and dispersal of metamorphs from the wetlands? Were adults sampled every year? How were individuals identified or marked? Some of this information can be gleaned from the Results, but aspects of the study that are under the control of the researcher and relevant to the manuscript should appear in the Materials and Methods.

In addition to providing additional information on the field methods, this manuscript would benefit greatly by providing the BUGS code (identified in the supporting R object as “VBGE2019.txt”) as supporting information. This code, or a figure providing the directed acyclic graph (DAG) of the model, would greatly enhance understanding of the linkage between the larval growth model, the metamorphic transition, and adult growth, and better enable researchers to use (and cite!) this paper in their own work. In addition to providing code, a description of how longevity was determined from these data and the model needs to be described in the analytical methods.

In addition to these general comments, I reference specific comments by line number below.

Specific Comments:

Line 55:	These statements about sampling methods for different amphibian stages are overly general. Although larval amphibians are typically sampled with nets or aquatic traps, I am unfamiliar with spotlight surveys for them. Even among terrestrial salamanders, sampling methods for adults can vary widely. The point about divergent methods for different life stages nonetheless is true for most amphibians with aquatic larvae and terrestrial adults.

Lines 71–78:	This paragraph would be better placed in the Materials and Methods.

Line 79:	A paragraph here about the objectives of the research or the research questions addressed would bring the broader context of the Introduction to a logical conclusion regarding the impetus for this specific study.

Lines 87–88:	See previous statement. It seems odd to reference the Introduction when referring to methods.

Line 106–112:	I recommend specifying the units for the parameters so that readers can better interpret the constraints imposed by the priors on lines 108–110.

Line 112:	I recommend indicating that *L_∞_* refers to the asymptotic size of adults across all presented von Bertalanffy growth models if that is the case.

Lines 115–117:	How is measurement error in this formulation separated from individual heterogeneity in growth, and how could these two sources of variation in *L_t_* be separated without marked individuals? It seems to me individual heterogeneity and measurement error would both be included in the variance parameter $\sigma_{L}^{2}$.

Line 134:	Is $L_{t_{m}}$ equivalent to *L_t_* on line 126? If so, I recommend changing *L_t_* on line 126 to $L_{t_{m}}$ to make this explicit.

Line 150:	It is probably worth citing the R2WinBUGS package here as well.

Lines 150–154:	This seems like a lot of thinning, resulting in only 12,000 samples from the posterior. What was the minimum effective sample size across parameters? Note that the posterior distributions provided in the supporting information are not based on these settings, but on a single chain of 3.5 million iterations, discarding the first 500,000 as burn-in, and thinned by a factor of 1000, yielding only 300 posterior samples.

Line 159:	Delete the “s” in “reductions.”

Line 167:	Replace “shows” with “showed” to keep the results in past tense.

Line 192:	I recommend replacing “parameterizations” with “life stages” to place the emphasis on the biology of flatwoods salamanders.

Lines 192–193:	The credible limits in Figure 4 appear to go from 20 mm to 50 mm. Why is the value in the text so much narrower? It almost seems like the dashed lines on the figure are the posterior predictive interval, rather than the credible interval. The same comment applies on line 195 for asymptotic length.

Line 201:	Replace “reflect” with “is.”

Lines 207–210:	How was longevity determined, given the plateau in size at 7 years, variability in individual size, and measurement error? A growth model with this shape seems ill-suited for estimating maximum lifespan; the mark-recapture data could, however, be used in other ways to try to estimate longevity (for an example, see Fellers et al. 2013).

Lines 254–264:	Better description of how the potential lifespan of flatwoods salamanders was estimated is necessary, otherwise this paragraph is largely speculative.

Lines 265–283:	This paragraph can be deleted. It is entirely based on unpublished data and a personal communication, and not on the model or results presented in this study.

Literature Cited:

Fellers, G. M., P. M. Kleeman, D. A. W. Miller, B. J. Halstead, and W. A. Link. 2013. Population size, survival, growth, and movements of *Rana sierrae*. Herpetologica 69:147–162.
