# Supplementary material for: Reconciling larval and adult sampling methods to model growth across life-stages
Source: PLoS One. 2020 Aug 21;15(8):e0237737. doi: 10.1371/journal.pone.0237737 (PMC7442236; doi:10.1371/journal.pone.0237737)
Supplement: Attachment — Submitted filename: PLOS rebuttal.docx [file pone.0237737.s003.docx]

**We would like to start by thanking the reviewers for their comments. I think they have greatly improved the manuscript. Following suggestions, we have provided more details of methods and sampling techniques, addressed reviewers’ comments to improve clarity and organization, and providing the BUGS code and raw data. Responses to specific comments follow.**

*1. Please ensure that your manuscript meets PLOS ONE's style requirements, including those for file naming.*

**We believe we have corrected any and all formatting errors**

*2. In your Methods section, please provide additional location information of the collection sites, including geographic coordinates for the data set if available.*

**We have added additional information about the study location, although specific GPS points cannot be revealed due to security concerns. (The study species is federally endangered and threatened with illegal collection for the pet trade.)**

*3a. Please amend your current ethics statement to include the full name of the ethics committee that approved your specific study.*

**corrected**

*3b. Once you have amended this/these statement(s) in the Methods section of the manuscript, please add the same text to the “Ethics Statement” field of the submission form*

**done**

4. In your Data Availability statement, you have not specified where the minimal data set underlying the results described in your manuscript can be found. PLOS defines a study's minimal data set as the underlying data used to reach the conclusions drawn in the manuscript and any additional data required to replicate the reported study findings in their entirety. All PLOS journals require that the minimal data set be made fully available. For more information about our data policy, please see <http://journals.plos.org/plosone/s/data-availability>.

**We have included the raw data as a supplementary file**

Reviewer #1:

*Overall, I thought this was an interesting manuscript that uses contemporary modeling methods to better understand growth and age in an imperiled vertebrate species. Authors provide a novel modeling framework that holds great utility, especially for researchers that study animals with complex life cycles. The research also has the potential to aid in endangered species recovery through a better understanding of life history.*
**Thanks! We think it will be useful too!**

*Abstract: Provide some results! Currently, results of quite general (i.e., marked variability in growth, the timing and age at metamorphosis, etc.)*

**We have added some specific results to the abstract**

*Line 27: Remove “hence”*
**done**

*Line 34: Topic sentence and supported sentences are mis-matched. The topic sentence indicates the paragraph will focus on the “many choices for those wishing to model population growth.” The supporting sentences do not focus on these model choices. Rather the focus is on “difficulty aging” and “techniques.”*
**we have revised the opening sentence to improve clarity**

*Line 40-41: Why are mark-recapture studies especially important for endangered species management? Is it related to non-lethal nature of capture-mark-recapture studies?*
**yes, as stated in the previous sentence of that paragraph**

*Line 49: Remove “however”*
**done**

*Line 87: I don’t recall reading details on drift fence design or protocols used. Did the driftnet completely encircle each pond(s)? When and how often were they checked? Spacing between buckets? How were animals marked? Are you using Total length or Snout-to-vent length?*

**we have added a description of the long-term drift fence study and methodologies.**

*Lines 92-97: I would think most readers understand the benefits of using a Bayesian hierarchical model. These benefits are sufficiently outlined by the authors. What isn’t clear, however, is if the authors are basing their model on the Eaton and Link model (i.e., citation 13). If so, an explanation of the Eaton and Link model is needed as well as any modification to this model. Additional information, such as the importance of accounting for measurement error, should also be highlighted.*

**The model is not based on Eaton and Link, so no description necessary, but a sentence highlighting the importance of measurement error has been added**

*Lines 101-157: I’m not an expert on growth models. However, I think the authors provide a decent explanation of their model. Choice of priors was not explained and may be needed?*
**We have added a sentence in the results addressing/explaining choice of priors**

*Lines 163-169: This paragraph should be the first one in the Results section as it explains the raw data as opposed to the modeling.*
**done**

*Lines 185: What sizes are needed to reach metamorphosis? Can you just provide the size?*
**included**

*Line 188-189: “Some evidence that size of metamorphosis impacted return rates the following year (Fig 3), possibly of reduced survival of smaller metamorphs”. First, I have difficulty interpreting this from Figure 3. Second, seems like this speculation (and lines 190-191) is best left for the discussion, if at all.*
**removed**

*Line 192: “…from larvae to adult..” Should this be larvae to metamorph?*
**corrected**

*Lines 206-207: “Similarly, discernment of the demographic structure…” Again, should this be in the discussion section?*
**removed**

*Lines 229-230: What are the sizes necessary to successfully transition across life-stages?*
**added clarification**

*Line 241 – remove “this” as it’s written twice*
**corrected**

*Line 255 – What are some of the ages obtained by other Ambystomatids? And survival estimates.*
**this is addressed in subsequent paragraphs in the discussion**

*Line 261 – The citation [51] is from Stearns, but it is being used as a comparison to related another species to flatwoods salamanders. This is not useful. Please be explicit in this comparison.*

**Removed erroneous citation**

*Line 265 – It is not clear if this topic sentence is based on your results? Who are the “individuals” you are discussing. Do the authors provide results that suggest distinct seasonal patterns? Where?*

**whole paragraph has been removed following reviewer 2 comment**


*Line 291 – remove “however”*
**whole paragraph has been removed following reviewer 2 comment**

Reviewer #2:

*In general, this manuscript was technically sound, but important details regarding field and some analytical methods were missing. The data were not available, which is understandable for such a rare species, but some other information, particularly the BUGS model code, would be very useful to readers.*

**Thanks! Yes, we definitely cannot release detailed site information owing to risk of illegal collection for the pet trade, but we can detail the field methods and include the BUGS code!**

*Line 55: These statements about sampling methods for different amphibian stages are overly general. Although larval amphibians are typically sampled with nets or aquatic traps, I am unfamiliar with spotlight surveys for them. Even among terrestrial salamanders, sampling methods for adults can vary widely. The point about divergent methods for different life stages nonetheless is true for most amphibians with aquatic larvae and terrestrial adults.*

**We have modified the sentence to improve clarity**

*Lines 71–78: This paragraph would be better placed in the Materials and Methods.*

**We have moved most of the paragraph to the methods section.**

*Line 79: A paragraph here about the objectives of the research or the research questions addressed would bring the broader context of the Introduction to a logical conclusion regarding the impetus for this specific study.*

**We have more clearly stated our objectives in the penultimate paragraph of the introduction**

*Lines 87–88: See previous statement. It seems odd to reference the Introduction when referring to methods.*

**We have included more detailed field methods**

*Line 106–112: I recommend specifying the units for the parameters so that readers can better interpret the constraints imposed by the priors on lines 108–110.*

**added**

Line 112: I recommend indicating that L ∞ refers to the asymptotic size of adults across all presented von Bertalanffy growth models if that is the case.

**added**

Lines 115–117: How is measurement error in this formulation separated from individual heterogeneity in growth, and how could these two sources of variation in L t be separated without marked individuals? It seems to me individual heterogeneity and measurement error would both be included in the variance parameter.

**Good catch! this should’ve been in the model for adult growth; we have moved it accordingly**

Line 134: Is equivalent to L t on line 126? If so, I recommend changing L t on line 126 to to make this explicit.

**corrected**

Line 150: It is probably worth citing the R2WinBUGS package here as well.

**done**

Line 159: Delete the “s” in “reductions.”

**Corrected**

Line 167: Replace “shows” with “showed” to keep the results in past tense.

**Corrected**

Line 192: I recommend replacing “parameterizations” with “life stages” to place the emphasis on the biology of flatwoods salamanders.

**Done**

Lines 192–193: The credible limits in Figure 4 appear to go from 20 mm to 50 mm. Why is the value in the text so much narrower? It almost seems like the dashed lines on the figure are the posterior predictive interval, rather than the credible interval. The same comment applies on line 195 for asymptotic length.

**Good catch! Corrected.**

Line 201: Replace “reflect” with “is.”

**Corrected**

*Lines 207–210: How was longevity determined, given the plateau in size at 7 years, variability in individual size, and measurement error? A growth model with this shape seems ill-suited for estimating maximum lifespan; the mark-recapture data could, however, be used in other ways to try to estimate longevity (for an example, see Fellers et al. 2013).*

**Yes there is a high degree of uncertainty in the age of larger individuals, and we agree this is not the best way to estimate longevity (longevity was more of a byproduct from the growth analysis). We have another manuscript in prep that is more focused on estimating survival rates / longevity explicitly as you suggest, however we feel this very rough estimate derived here from the growth increments is worth including, if only to act as a straw man for subsequent analysis. We have shortened this section so that is not as prominent in the manuscript.**

Lines 254–264: Better description of how the potential lifespan of flatwoods salamanders was estimated is necessary, otherwise this paragraph is largely speculative.

**Added details for clarity**

Lines 265–283: This paragraph can be deleted. It is entirely based on unpublished data and a personal communication, and not on the model or results presented in this study.

**removed**
