# Supplementary material for: Reconciling larval and adult sampling methods to model growth across life-stages
Source: PLoS One. 2020 Aug 21;15(8):e0237737. doi: 10.1371/journal.pone.0237737 (PMC7442236; doi:10.1371/journal.pone.0237737)
Supplement: Attachment — Submitted filename: PLOS rebuttal 2.docx [file pone.0237737.s004.docx]

**We would like to start by thanking the reviewers for their comments. I think they have greatly improved the manuscript. We apologize for missing these general comments in the first round of revisions.**

*This manuscript addresses two important issues related to modeling population growth of organisms with complex life-histories: 1) the inability to accurately age individuals of many species, and 2) quantifying changes in growth across life stages of such organisms. The manuscript presents a model that provides estimates of parameters from larval growth through metamorphosis to adult growth. Such a model is very appealing, because it allows estimation of growth parameters across life-history stages to encompass an organism’s entire lifespan. Being able to model growth across life stages can be particularly important for parameterizing population models to develop conservation strategies. Thus, the topic of this manuscript is both interesting and important.*

**Thanks, we think so too!**

*Although the authors have improved the Materials and Methods by providing many of the details regarding the drift fence sampling, some additional information is necessary. In particular, for studies that rely on recaptures of individuals, it is important to indicate how individuals identified or marked.*

**We have added the marking method**

*Also, because of the centrality of size measurements for a growth study, it is important to indicate how larvae and adults were measured for readers to assess whether it is appropriate to share an error term across life stages in the model.*
**We have added the measuring method**

*Specific Comments:
Lines 78: I recommend replacing the comma with “and” because the structure of the last element of the list (“variability in the timing and size at metamorphosis”) results in only two elements.*
**corrected**

*Line 89: Delete the period after “cm” and begin “funnel” with a lower case “f” if the preceding fragment is the size of the funnel traps.*
**good catch! corrected**

*Line 96: How was SVL measured? Was the same measurement method used for larvae (following paragraph)? I recommend including these details as they are relevant when trying to model growth across life-stage transitions.*
**added measuring methods**

*Lines 122, 136, & 156: The equations on these lines appear to be missing a negative sign in front of the growth coefficient. It correctly appears on line 145.*
**corrected**

*Lines 138–139: tm is technically not the age at metamorphosis, but the time since the individual was theoretically length 0, correct? Is that incorporated into the prior, and is interpretation and subsequent use (e.g., in the adult growth equations) of tm as age at metamorphosis adjusted for the difference between hatching date and theoretical age at length 0? Reporting the posterior distribution of t0 also would help to explain how influential this technicality is: the closer t0 is to 0, the more closely tm will correspond to the age at metamorphosis.*
**The posterior distribution for t0 was very narrow, and approximately centered on zero, so doesn’t unduly influence interpretation. Despite this, we retained t0 in the adult growth equations for completeness. We have added a sentence to the results clarifying this.**

*Line 161: It is unclear to me how σ_L^2 separates measurement error from individual variation in growth. It seems to me that this error term would account for any variation between the model-estimated size, Lt, and the observed length, Lobs. Yes, this would include measurement error, but would it not also include individual heterogeneity? Even if individuals were measured perfectly, one would expect that Lobs ≠ Lt, as is the case for residual error when nearly any model is model fitted to data.*
**you are correct; this is a relict from when we actually thought we could partition these two sources of uncertainty. I have clarified this where relevant.**

*Lines 166–169: If I am reading this correctly, the number of retained posterior samples was 400,000 x 3 / 100 = 12,000. What was the minimum effective sample size across sampled parameters?*

**The lowest ESS across parameters was approximately 600. I have added a sentence to the results stating this.**
